# *trans*-Dichloridobis[tris­(2-methoxy­phen­yl)phosphine]palladium(II)

**DOI:** 10.1107/S1600536809040719

**Published:** 2009-10-10

**Authors:** Charmaine van Blerk, Cedric W. Holzapfel

**Affiliations:** a University of Johannesburg, Department of Chemistry, PO Box 524, Auckland Park, Johannesburg 2006, South Africa

## Abstract

The structure of the title compound, [PdCl_2_(C_21_H_21_O_3_P)_2_], shows a nearly square-planar geometry for the Pd^II^ atom within the Cl_2_Pd[P(PhOMe)_3_]_2_ ligand set. The Pd^II^ atom sits on a centre of inversion and therefore the asymmetric unit contains one half-mol­ecule, *i.e.* half of one Pd^II^ atom, one Cl atom and one tris­(2-methoxy­phen­yl)phosphine ligand.

## Related literature

For related structures and literature on similar palladium complexes, see: Robertson & Cole-Hamilton (2002 ▶); Van Leeuwen *et al.* (2003 ▶); Williams *et al.* (2008 ▶).
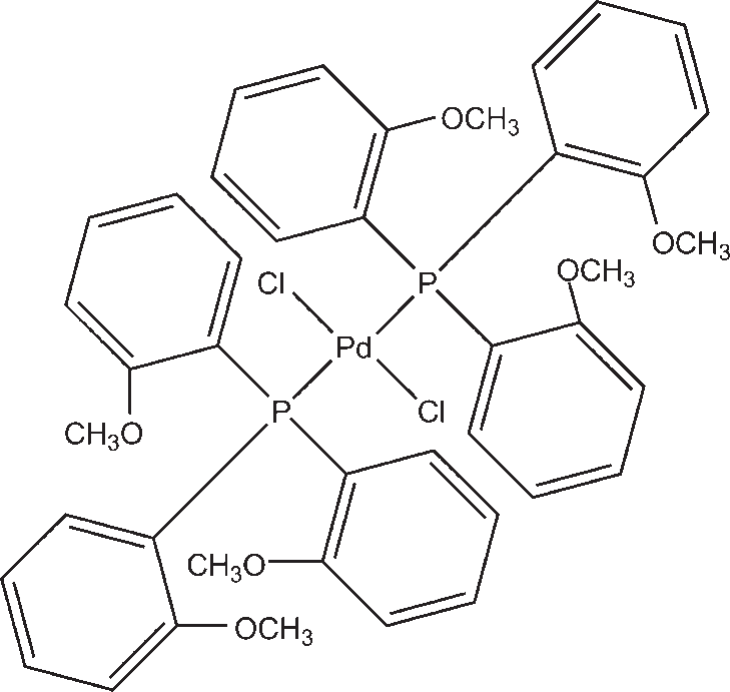


## Experimental

### 

#### Crystal data

[PdCl_2_(C_21_H_21_O_3_P)_2_]
*M* *_r_* = 882.00
Triclinic, 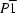

*a* = 9.1415 (2) Å
*b* = 10.8985 (3) Å
*c* = 12.0287 (3) Å
α = 103.691 (2)°
β = 109.275 (3)°
γ = 108.438 (2)°
*V* = 992.26 (5) Å^3^
*Z* = 1
Mo *K*α radiation
μ = 0.73 mm^−1^
*T* = 294 K
0.30 × 0.16 × 0.10 mm

#### Data collection

Bruker SMART CCD diffractometer
Absorption correction: multi-scan (*APEX2 AXScale*; Bruker, 2008 ▶) *T*_min_ = 0.811, *T*_max_ = 0.931
11296 measured reflections
4894 independent reflections
3672 reflections with *I* > 2σ(*I*)
*R*_int_ = 0.039

#### Refinement

*R*[*F* ^2^ > 2σ(*F* ^2^)] = 0.037
*wR*(*F* ^2^) = 0.094
*S* = 0.97
4894 reflections
244 parameters
H-atom parameters constrained
Δρ_max_ = 0.35 e Å^−3^
Δρ_min_ = −0.43 e Å^−3^

### 

Data collection: *SMART-NT* (Bruker, 1999 ▶); cell refinement: *SAINT* (Bruker, 2008 ▶); data reduction: *SAINT*; program(s) used to solve structure: *SHELXS97* (Sheldrick, 2008 ▶); program(s) used to refine structure: *SHELXL97* (Sheldrick, 2008 ▶); molecular graphics: *X-SEED* (Barbour, 2001 ▶) and *Mercury* (Macrae *et al.*, 2006 ▶); software used to prepare material for publication: *publCIF* (Westrip, 2009 ▶).

## Supplementary Material

Crystal structure: contains datablocks I, global. DOI: 10.1107/S1600536809040719/bq2163sup1.cif

Structure factors: contains datablocks I. DOI: 10.1107/S1600536809040719/bq2163Isup2.hkl

Additional supplementary materials:  crystallographic information; 3D view; checkCIF report

## Figures and Tables

**Table:** 

Pd1—Cl1	2.3120 (7)
Pd1—P1	2.3417 (7)

**Table:** 

Cl1—Pd1—P1^i^	94.27 (3)
Cl1—Pd1—P1	85.73 (3)

Symmetry code: (i) 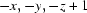.

**Fig. 1.**
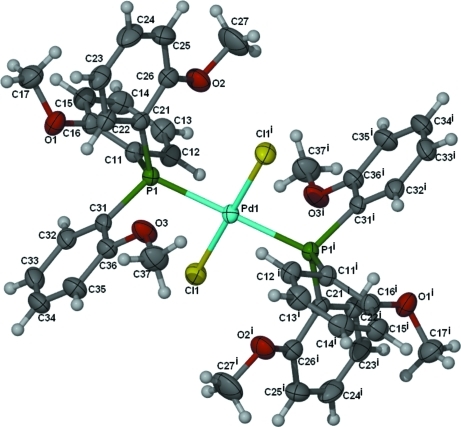
: Molecular structure of (I) with displacement ellipsoids drawn at the 50% probability level.[Symmetry code:(i) -x, -y, -z+1].

## supplementary crystallographic information

### Comment

The palladium-catalysed methoxycarbonylation (Robertson and Cole-Hamilton, 2002) of 1-alkenes is an active area of research. The preferred (pre)-catalysts of general structure (Ar_3_P)_2_Pd*X*_2_ (*X* = Cl, DMS, OTf *etc*.) are either preformed or generated *in situ*. The *x*-ray structures (Van Leeuwen *et al.*, 2003 and Williams *et al.*, 2008) of several of this class of palladium(II) complexes have been determined. Only some of these have found application in the catalysis of the methoxycarbonylation reaction, but their use results mainly in the formation of linear esters (Robertson and Cole-Hamilton, 2002). However, we have identified some palladium(II) complexes which catalyse the regioselective formation of branched esters. We report here the structure of one of these.

The structure of the title compound, [PdCl_2_(C_42_H_42_P_2_O_6_)], (I), shows a nearly square planar geometry (Table 1.) for the Pd^II^ atom within the Cl_2_(P(PhOMe)_3_) ligand set. The palladium atom sits on a centre of inversion and therefore the the asymmetric unit contains the half of the molecule, i.e. half of the palladium atom, one chlorine atom and one tris-(2-methoxyphenyl)phosphine ligand (Figure 1.)

### Experimental

Starting ligand material, tris-(2-methoxyphenyl)phosphine (1.408 g, 4 mmol) was added to a solution of palladium(II) chloride (1.288 g, 2 mmol) and anhydrous lithium chloride (168 mg, 4 mmol) in 15 ml methanol. The mixture was stirred under reflux in an atmosphere of nitrogen until all the phosphine reagent had reacted and a yellow product had formed (*ca* 1 hr). The reaction mixture was cooled and the product collected by filtration; washed with fresh methanol and dried under vacuum. The crude product (1.41 g) was dissolved in dichloromethane and crystallization of the title compound was carried out by diethyl ether vapor diffusion into the dichloromethane. The crystals of the title compound were bright yellow prisms (m. p. > 222° C, decomp.) and a suitable crystal was selected for the single-crystal X-ray diffraction analysis.

### Refinement

H atoms were geometrically positioned and refined in the riding-model approximation, with C—H = 0.93-0.96 Å, and *U*_iso_(H) = 1.2Ueq(C) or 1.5Ueq(C for Me). For (I), the highest peak in the final difference map is 1.01 Å from Pd1 and the deepest hole is 0.01 Å from Pd1.

### Crystal data

**Table:**

[PdCl_2_(C_42_H_42_O_6_P_2_)]	*Z* = 1
*M**_r_* = 882.00	*F*(000) = 452
Triclinic, *P*1	*D*_x_ = 1.476 Mg m^−^^3^
Hall symbol: -P 1	Mo *K*α radiation, λ = 0.71073 Å
*a* = 9.1415 (2) Å	Cell parameters from 3513 reflections
*b* = 10.8985 (3) Å	θ = 2.3–25.9°
*c* = 12.0287 (3) Å	µ = 0.73 mm^−^^1^
α = 103.691 (2)°	*T* = 294 K
β = 109.275 (3)°	Plate, yellow
γ = 108.438 (2)°	0.30 × 0.16 × 0.10 mm
*V* = 992.26 (5) Å^3^	

### Data collection

**Table:**

Bruker SMART CCD diffractometer	4894 independent reflections
Radiation source: fine-focus sealed tube	3672 reflections with *I* > 2σ(*I*)
graphite	*R*_int_ = 0.039
φ and ω scans	θ_max_ = 28.3°, θ_min_ = 1.9°
Absorption correction: multi-scan (*APEX2* AXScale; Bruker, 2008)	*h* = −12→11
*T*_min_ = 0.811, *T*_max_ = 0.931	*k* = −14→13
11296 measured reflections	*l* = −15→16

### Refinement

**Table:**

Refinement on *F*^2^	Primary atom site location: structure-invariant direct methods
Least-squares matrix: full	Secondary atom site location: difference Fourier map
*R*[*F*^2^ > 2σ(*F*^2^)] = 0.037	Hydrogen site location: inferred from neighbouring sites
*wR*(*F*^2^) = 0.094	H-atom parameters constrained
*S* = 0.97	*w* = 1/[σ^2^(*F*_o_^2^) + (0.051*P*)^2^] where *P* = (*F*_o_^2^ + 2*F*_c_^2^)/3
4894 reflections	(Δ/σ)_max_ < 0.001
244 parameters	Δρ_max_ = 0.35 e Å^−^^3^
0 restraints	Δρ_min_ = −0.43 e Å^−^^3^

### Special details

**Table:**

Geometry. All e.s.d.'s (except the e.s.d. in the dihedral angle between two l.s. planes) are estimated using the full covariance matrix. The cell e.s.d.'s are taken into account individually in the estimation of e.s.d.'s in distances, angles and torsion angles; correlations between e.s.d.'s in cell parameters are only used when they are defined by crystal symmetry. An approximate (isotropic) treatment of cell e.s.d.'s is used for estimating e.s.d.'s involving l.s. planes.
Refinement. Refinement of *F*^2^ against ALL reflections. The weighted *R*-factor *wR* and goodness of fit *S* are based on *F*^2^, conventional *R*-factors *R* are based on *F*, with *F* set to zero for negative *F*^2^. The threshold expression of *F*^2^ > σ(*F*^2^) is used only for calculating *R*-factors(gt) *etc*. and is not relevant to the choice of reflections for refinement. *R*-factors based on *F*^2^ are statistically about twice as large as those based on *F*, and *R*- factors based on ALL data will be even larger.

### Fractional atomic coordinates and isotropic or equivalent isotropic displacement parameters (Å^2^)

**Table:**

	*x*	*y*	*z*	*U*_iso_*/*U*_eq_	
Pd1	0.0000	0.0000	0.5000	0.02713 (10)	
Cl1	0.15837 (9)	−0.10437 (8)	0.59870 (7)	0.04185 (18)	
P1	−0.08161 (8)	−0.18084 (7)	0.30980 (7)	0.02821 (16)	
O1	−0.0797 (3)	−0.3741 (2)	0.0829 (2)	0.0456 (5)	
O2	−0.0304 (3)	0.0196 (2)	0.1933 (2)	0.0514 (6)	
O3	−0.3775 (3)	−0.2921 (2)	0.3590 (2)	0.0499 (6)	
C11	0.0985 (3)	−0.1701 (3)	0.2718 (3)	0.0330 (6)	
C12	0.2580 (4)	−0.0560 (3)	0.3521 (3)	0.0389 (7)	
H12	0.2748	0.0032	0.4295	0.047*	
C13	0.3914 (4)	−0.0308 (3)	0.3165 (3)	0.0460 (8)	
H13	0.4969	0.0453	0.3702	0.055*	
C14	0.3682 (4)	−0.1176 (4)	0.2026 (3)	0.0477 (8)	
H14	0.4581	−0.1002	0.1796	0.057*	
C15	0.2113 (4)	−0.2308 (3)	0.1219 (3)	0.0449 (8)	
H15	0.1958	−0.2890	0.0446	0.054*	
C16	0.0765 (4)	−0.2577 (3)	0.1565 (3)	0.0367 (7)	
C17	−0.1525 (5)	−0.4108 (4)	−0.0510 (3)	0.0573 (9)	
H17A	−0.1702	−0.3344	−0.0698	0.086*	
H17B	−0.2605	−0.4919	−0.0906	0.086*	
H17C	−0.0761	−0.4309	−0.0832	0.086*	
C21	−0.2402 (3)	−0.1916 (3)	0.1633 (3)	0.0321 (6)	
C22	−0.4005 (4)	−0.3027 (3)	0.0900 (3)	0.0374 (7)	
H22	−0.4308	−0.3776	0.1153	0.045*	
C23	−0.5172 (4)	−0.3056 (4)	−0.0200 (3)	0.0471 (8)	
H23	−0.6235	−0.3822	−0.0693	0.056*	
C24	−0.4724 (5)	−0.1919 (4)	−0.0553 (3)	0.0572 (9)	
H24	−0.5514	−0.1908	−0.1270	0.069*	
C25	−0.3139 (5)	−0.0814 (4)	0.0140 (3)	0.0540 (9)	
H25	−0.2853	−0.0065	−0.0115	0.065*	
C26	−0.1954 (4)	−0.0806 (3)	0.1223 (3)	0.0388 (7)	
C27	0.0186 (6)	0.1455 (4)	0.1722 (5)	0.0838 (14)	
H27A	−0.0617	0.1842	0.1745	0.126*	
H27B	0.0196	0.1268	0.0904	0.126*	
H27C	0.1313	0.2110	0.2374	0.126*	
C31	−0.1653 (3)	−0.3488 (3)	0.3249 (3)	0.0311 (6)	
C32	−0.0820 (4)	−0.4361 (3)	0.3219 (3)	0.0397 (7)	
H32	0.0107	−0.4142	0.3021	0.048*	
C33	−0.1362 (5)	−0.5554 (3)	0.3482 (3)	0.0507 (9)	
H33	−0.0805	−0.6135	0.3454	0.061*	
C34	−0.2717 (5)	−0.5873 (3)	0.3784 (3)	0.0574 (9)	
H34	−0.3069	−0.6668	0.3969	0.069*	
C35	−0.3573 (5)	−0.5032 (3)	0.3817 (3)	0.0522 (9)	
H35	−0.4505	−0.5267	0.4009	0.063*	
C36	−0.3034 (4)	−0.3833 (3)	0.3561 (3)	0.0403 (7)	
C37	−0.5075 (5)	−0.3122 (4)	0.4017 (4)	0.0604 (10)	
H37A	−0.4612	−0.3054	0.4882	0.091*	
H37B	−0.6007	−0.4031	0.3488	0.091*	
H37C	−0.5486	−0.2418	0.3968	0.091*	

### Atomic displacement parameters (Å^2^)

**Table:**

	*U*^11^	*U*^22^	*U*^33^	*U*^12^	*U*^13^	*U*^23^
Pd1	0.02580 (16)	0.02587 (16)	0.02807 (18)	0.01139 (13)	0.01105 (13)	0.00837 (13)
Cl1	0.0434 (4)	0.0428 (4)	0.0399 (4)	0.0256 (4)	0.0126 (4)	0.0151 (4)
P1	0.0270 (4)	0.0260 (4)	0.0292 (4)	0.0104 (3)	0.0123 (3)	0.0080 (3)
O1	0.0460 (13)	0.0431 (12)	0.0391 (13)	0.0129 (11)	0.0218 (11)	0.0058 (11)
O2	0.0507 (14)	0.0417 (12)	0.0597 (16)	0.0118 (11)	0.0254 (12)	0.0255 (12)
O3	0.0570 (14)	0.0500 (13)	0.0703 (17)	0.0293 (12)	0.0437 (13)	0.0363 (13)
C11	0.0313 (14)	0.0336 (15)	0.0346 (16)	0.0148 (12)	0.0158 (13)	0.0107 (13)
C12	0.0347 (16)	0.0396 (16)	0.0374 (17)	0.0140 (14)	0.0153 (14)	0.0104 (14)
C13	0.0309 (16)	0.0493 (19)	0.056 (2)	0.0150 (15)	0.0182 (16)	0.0205 (17)
C14	0.0445 (18)	0.060 (2)	0.060 (2)	0.0299 (17)	0.0343 (18)	0.0301 (19)
C15	0.0510 (19)	0.0494 (19)	0.045 (2)	0.0264 (17)	0.0296 (17)	0.0175 (17)
C16	0.0381 (16)	0.0345 (15)	0.0401 (18)	0.0169 (13)	0.0199 (14)	0.0128 (14)
C17	0.062 (2)	0.055 (2)	0.040 (2)	0.0152 (19)	0.0176 (18)	0.0142 (18)
C21	0.0349 (15)	0.0357 (15)	0.0280 (15)	0.0174 (13)	0.0150 (13)	0.0111 (13)
C22	0.0354 (16)	0.0389 (16)	0.0338 (16)	0.0168 (14)	0.0134 (14)	0.0089 (14)
C23	0.0415 (18)	0.053 (2)	0.0350 (18)	0.0240 (16)	0.0079 (15)	0.0063 (16)
C24	0.061 (2)	0.073 (3)	0.0312 (18)	0.039 (2)	0.0069 (17)	0.0153 (18)
C25	0.076 (3)	0.054 (2)	0.044 (2)	0.035 (2)	0.026 (2)	0.0282 (18)
C26	0.0434 (17)	0.0390 (16)	0.0357 (17)	0.0185 (14)	0.0191 (15)	0.0135 (14)
C27	0.092 (3)	0.052 (2)	0.102 (4)	0.013 (2)	0.041 (3)	0.047 (3)
C31	0.0337 (14)	0.0236 (13)	0.0253 (14)	0.0084 (12)	0.0070 (12)	0.0056 (12)
C32	0.0413 (17)	0.0349 (16)	0.0309 (16)	0.0145 (14)	0.0073 (14)	0.0079 (13)
C33	0.067 (2)	0.0354 (17)	0.042 (2)	0.0262 (17)	0.0112 (18)	0.0138 (16)
C34	0.082 (3)	0.0341 (18)	0.047 (2)	0.0177 (18)	0.021 (2)	0.0208 (17)
C35	0.064 (2)	0.0405 (18)	0.056 (2)	0.0166 (17)	0.0304 (19)	0.0270 (18)
C36	0.0459 (17)	0.0335 (15)	0.0386 (18)	0.0135 (14)	0.0178 (15)	0.0153 (14)
C37	0.064 (2)	0.067 (2)	0.073 (3)	0.030 (2)	0.048 (2)	0.036 (2)

### Geometric parameters (Å, °)

**Table:**

Pd1—Cl1^i^	2.3120 (7)	C21—C22	1.382 (4)
Pd1—Cl1	2.3120 (7)	C21—C26	1.406 (4)
Pd1—P1^i^	2.3417 (7)	C22—C23	1.385 (4)
Pd1—P1	2.3417 (7)	C22—H22	0.9300
P1—C11	1.826 (3)	C23—C24	1.388 (5)
P1—C31	1.825 (3)	C23—H23	0.9300
P1—C21	1.824 (3)	C24—C25	1.367 (5)
O1—C16	1.386 (3)	C24—H24	0.9300
O1—C17	1.420 (4)	C25—C26	1.389 (4)
O2—C26	1.365 (4)	C25—H25	0.9300
O2—C27	1.415 (4)	C27—H27A	0.9600
O3—C36	1.370 (4)	C27—H27B	0.9600
O3—C37	1.419 (4)	C27—H27C	0.9600
C11—C16	1.394 (4)	C31—C32	1.395 (4)
C11—C12	1.400 (4)	C31—C36	1.398 (4)
C12—C13	1.391 (4)	C32—C33	1.388 (4)
C12—H12	0.9300	C32—H32	0.9300
C13—C14	1.373 (5)	C33—C34	1.368 (5)
C13—H13	0.9300	C33—H33	0.9300
C14—C15	1.384 (5)	C34—C35	1.383 (5)
C14—H14	0.9300	C34—H34	0.9300
C15—C16	1.395 (4)	C35—C36	1.388 (4)
C15—H15	0.9300	C35—H35	0.9300
C17—H17A	0.9600	C37—H37A	0.9600
C17—H17B	0.9600	C37—H37B	0.9600
C17—H17C	0.9600	C37—H37C	0.9600
			
Cl1^i^—Pd1—Cl1	180.00 (4)	C21—C22—H22	119.1
Cl1^i^—Pd1—P1^i^	85.73 (3)	C22—C23—C24	118.7 (3)
Cl1—Pd1—P1^i^	94.27 (3)	C22—C23—H23	120.7
Cl1^i^—Pd1—P1	94.27 (3)	C24—C23—H23	120.7
Cl1—Pd1—P1	85.73 (3)	C25—C24—C23	120.9 (3)
P1^i^—Pd1—P1	180.0	C25—C24—H24	119.5
C11—P1—C31	107.32 (13)	C23—C24—H24	119.5
C11—P1—C21	102.23 (13)	C24—C25—C26	120.2 (3)
C31—P1—C21	107.11 (13)	C24—C25—H25	119.9
C11—P1—Pd1	112.29 (9)	C26—C25—H25	119.9
C31—P1—Pd1	109.33 (9)	O2—C26—C25	124.9 (3)
C21—P1—Pd1	117.89 (9)	O2—C26—C21	115.1 (3)
C16—O1—C17	117.7 (2)	C25—C26—C21	120.0 (3)
C26—O2—C27	119.1 (3)	O2—C27—H27A	109.5
C36—O3—C37	119.0 (2)	O2—C27—H27B	109.5
C16—C11—C12	118.9 (2)	H27A—C27—H27B	109.5
C16—C11—P1	122.1 (2)	O2—C27—H27C	109.5
C12—C11—P1	118.1 (2)	H27A—C27—H27C	109.5
C13—C12—C11	120.2 (3)	H27B—C27—H27C	109.5
C13—C12—H12	119.9	C32—C31—C36	118.6 (3)
C11—C12—H12	119.9	C32—C31—P1	121.6 (2)
C14—C13—C12	120.4 (3)	C36—C31—P1	119.3 (2)
C14—C13—H13	119.8	C33—C32—C31	120.7 (3)
C12—C13—H13	119.8	C33—C32—H32	119.7
C13—C14—C15	120.2 (3)	C31—C32—H32	119.7
C13—C14—H14	119.9	C34—C33—C32	119.8 (3)
C15—C14—H14	119.9	C34—C33—H33	120.1
C14—C15—C16	120.1 (3)	C32—C33—H33	120.1
C14—C15—H15	120.0	C33—C34—C35	120.9 (3)
C16—C15—H15	120.0	C33—C34—H34	119.5
O1—C16—C11	117.3 (2)	C35—C34—H34	119.5
O1—C16—C15	122.4 (3)	C34—C35—C36	119.6 (3)
C11—C16—C15	120.2 (3)	C34—C35—H35	120.2
O1—C17—H17A	109.5	C36—C35—H35	120.2
O1—C17—H17B	109.5	O3—C36—C35	124.1 (3)
H17A—C17—H17B	109.5	O3—C36—C31	115.5 (2)
O1—C17—H17C	109.5	C35—C36—C31	120.4 (3)
H17A—C17—H17C	109.5	O3—C37—H37A	109.5
H17B—C17—H17C	109.5	O3—C37—H37B	109.5
C22—C21—C26	118.3 (3)	H37A—C37—H37B	109.5
C22—C21—P1	123.9 (2)	O3—C37—H37C	109.5
C26—C21—P1	117.8 (2)	H37A—C37—H37C	109.5
C23—C22—C21	121.8 (3)	H37B—C37—H37C	109.5
C23—C22—H22	119.1		

Symmetry codes: (i) −*x*, −*y*, −*z*+1.
